# Use of Expansion Factors to Estimate the Burden of Dengue in Southeast Asia: A Systematic Analysis

**DOI:** 10.1371/journal.pntd.0002056

**Published:** 2013-02-21

**Authors:** Eduardo A. Undurraga, Yara A. Halasa, Donald S. Shepard

**Affiliations:** Schneider Institutes for Health Policy, Heller School for Social Policy and Management, Brandeis University, Waltham, Massachusetts, United States of America; Pediatric Dengue Vaccine Initiative, United States of America

## Abstract

**Background:**

Dengue virus infection is the most common arthropod-borne disease of humans and its geographical range and infection rates are increasing. Health policy decisions require information about the disease burden, but surveillance systems usually underreport the total number of cases. These may be estimated by multiplying reported cases by an expansion factor (EF).

**Methods and Findings:**

As a key step to estimate the economic and disease burden of dengue in Southeast Asia (SEA), we projected dengue cases from 2001 through 2010 using EFs. We conducted a systematic literature review (1995–2011) and identified 11 published articles reporting original, empirically derived EFs or the necessary data, and 11 additional relevant studies. To estimate EFs for total cases in countries where no empirical studies were available, we extrapolated data based on the statistically significant inverse relationship between an index of a country's health system quality and its observed reporting rate. We compiled an average 386,000 dengue episodes reported annually to surveillance systems in the region, and projected about 2.92 million dengue episodes. We conducted a probabilistic sensitivity analysis, simultaneously varying the most important parameters in 20,000 Monte Carlo simulations, and derived 95% certainty level of 2.73–3.38 million dengue episodes. We estimated an overall EF in SEA of 7.6 (95% certainty level: 7.0–8.8) dengue cases for every case reported, with an EF range of 3.8 for Malaysia to 19.0 in East Timor.

**Conclusion:**

Studies that make no adjustment for underreporting would seriously understate the burden and cost of dengue in SEA and elsewhere. As the sites of the empirical studies we identified were not randomly chosen, the exact extent of underreporting remains uncertain. Nevertheless, the results reported here, based on a systematic analysis of the available literature, show general consistency and provide a reasonable empirical basis to adjust for underreporting.

## Author Summary

Dengue is the most common disease transmitted by a mosquito, with about 100–200 million infections occurring each year in more than 100 tropical and subtropical countries. Policy-makers require accurate information about the number of symptomatic dengue episodes to make informed decisions concerning dengue control strategies. But dengue is usually underreported by national surveillance systems. Through a systematic literature review and analysis of empirical research, we estimated the rate of underreporting and the average annual dengue episodes by treatment (hospitalized and ambulatory) in 2001–2010, for 12 countries in Southeast Asia. We found an average reporting rate of 13.2% of the total symptomatic dengue episodes in the region, leading to an expansion factor of 7.6 for converting reported cases into estimated actual cases. While we focused in Southeast Asia, analogous principles apply to other regions of the world, and other diseases reported through surveillance systems. Estimating the total episodes of dengue is a critical step in studying the economic and disease burden of dengue fever, and the cost-effectiveness evaluation of dengue control and prevention strategies.

## Introduction

Dengue has become a major public health problem in many tropical and subtropical regions, with an estimate of 100–200 million dengue infections occurring each year in more than 100 countries, resulting in approximately 20,000 deaths [1]. It represents a significant economic burden to communities and health services in endemic countries, with a thirtyfold incidence increase in the last fifty years [2]. A variety of factors have created the ideal conditions for the expansion and distribution of dengue mosquito vector and viruses, including high rates of population growth, inadequate water, sewer, and waste management systems, rise in global commerce and tourism, global warming, changes in public health policy, and the development of hyperendimicity in urban areas, although it is difficult to estimate the contributions of each factor separately [3]–[9].

Southeast Asia has the world's largest incidence of dengue, with cycles of epidemics of increasing magnitude occurring every three to five years [6], [10]. The WHO regions of Southeast Asia and Western Pacific represent most of the current global burden of dengue [11], and account for most deaths [12]. All four dengue serotypes have been found in most countries of SEA [13] which means that one person can get up to four dengue infections. The risk of developing a more severe manifestation of dengue illness (e.g., dengue hemorrhagic fever (DHF) and dengue shock syndrome, DSS) increases with subsequent infections [14], [15], which may explain the higher frequency of DHF and DSS in hyperendemic countries, such as those in SEA.

Having accurate information about the human and economic burden of dengue is essential to inform policy makers and international donors, set health policy priorities, and make informed decisions about disease-control technologies and resource allocation. Estimating the total cases of symptomatic dengue infection is a critical step in calculating its economic and disease burden, but dengue incidence data are heterogeneous and incomplete. Most dengue episodes are identified using clinical diagnosis and existing epidemiological information, and, less frequently, clinical laboratory tests [13]. Passive surveillance systems require that identified episodes of dengue fever be reported, usually to the Ministry of Health (MoH), but reported episodes do not consistently indicate severity of dengue. While passive surveillance systems, which rely on clinicians' reports incidental to their providing treatment, are appropriate to help detect dengue outbreaks promptly and examine long-term trends, they are not designed to estimate the real disease burden and usually underreport the total number of symptomatic dengue cases [13], [16]–[20]. Limitations of passive surveillance systems include variations in both the system design and in the characteristics of surveillance implementation. Variations in system design include dengue case definition, clinical or lab-confirmed diagnosis, inpatient and outpatient reporting, reporting from sentinel or all hospitals and health services, public and private sector reporting, specific ages or dengue severity reported, and surveillance budget. The characteristics of surveillance implementation include reliance on health care professionals and laboratory staff, use of electronic or paper forms, and people's health-seeking behavior. Other sources of variation of dengue reporting rates include whether data is collected on epidemic or non-epidemic years, unrecognized or mild symptoms, the overall quality of the health care system, and the specific area of the country where incidence of dengue is measured (e.g., rural or urban) [6], [13], [17], [21]–[26].

While Singapore has recently implemented more sophisticated techniques to report cases and promptly respond to dengue epidemics [24], [26], and Malaysia has some enhanced capacity [24], most countries in SEA have only passive surveillance systems [25], making underreporting of dengue episodes a significant challenge in estimating disease burden. Based on a systematic literature review and using available data from surveillance systems, we estimated the average annual episodes of dengue by type of treatment in 12 countries from SEA (2001–2010). We defined SEA for this study as consisting of the following 12 countries: Bhutan, Brunei, Cambodia, East-Timor, Indonesia, Laos, Malaysia, Myanmar, Philippines, Singapore, Thailand, and Viet Nam. We wanted to focus on a contiguous area with hyperendemic dengue and a reasonable amount of available data. We included all countries in the Association of Southeast Asian Nations, as in a previous cost-effectiveness study [27], and added the bordering countries Bhutan and East-Timor due to their geographic proximity [28]. We obtained the incidence of symptomatic dengue by adjusting the reported episodes using an “expansion factor” (EF), which corrected for underreporting. While total dengue episodes remain an area of considerable uncertainty, we used the best empirical data available to provide as accurate estimates as possible.

## Methods

### Data sources, parameters, and strategy

We combined various data sources to obtain our best estimate of the average annual episodes of dengue in 2001–2010. The data included a systematic literature review of articles (1995–2012) that reported empirically derived EFs or the necessary data to estimate them, and available surveillance data on reported dengue episodes. An EF is the number by which reported cases need to be multiplied to obtain the most accurate estimate of the true number of episodes The goal was to obtain total episodes by identifying the following parameters: reported episodes, EFs for total (EF_T_: total episodes/reported episodes), hospitalized (EF_H_: total hospitalized episodes/reported hospitalized episodes), and ambulatory cases (EF_A_: total ambulatory episodes/reported ambulatory episodes) where possible, and last, the outpatient to inpatient ratio (OP: IP) to allow extrapolation from hospitalized episodes to total episodes, where necessary. Because the availability of country-specific data and the quality of published studies varied substantially, we used the best data available and extrapolated parameters based on a measure of health system quality and on assumptions about reporting of hospitalized episodes and OP∶IP (discussed below).

### Reported cases

We obtained the reported cases of dengue from various sources, including surveillance data from country-specific MoH or department of statistics [29]–[34] and WHO regional offices [11], [13], . Cambodia was the only country considered in this study that only reported dengue episodes affecting patients of less than 15 years old [17], [26], [38]. Because dengue is an infectious disease, the number of cases varied considerably among years. To generate more stable estimates of the total projected cases of dengue, we considered the average reported cases in the last decade of available data (2001–2010).

### Estimates of expansion factors and total dengue episodes in Southeast Asia

An EF can be calculated as the analyst's best estimate of the total number of dengue episodes in a specified population divided by the episodes reported (whether or not they actually were laboratory-confirmed dengue). A recent study of the economic impact of dengue in the Americas identified five studies that permitted estimating EFs for the reported episodes of dengue [16]. The EFs ranged from 1.6 inpatient dengue episodes reported for each hospitalized episode in Brazil (1996–2002) [39] to 28 episodes of dengue for each clinically diagnosed episode in Nicaragua (2005–2006) [40].

While there are considerable regional differences in the epidemiology of dengue between SEA and the Americas, in both regions dengue consists of the same four virus serotypes, is officially notifiable, and is considerably underreported [41]. The attack rates of DHF and DSS in SEA are approximately 18 times that of the Americas, with infants and children most affected [41]. Some authors have used EFs obtained from studies in the Americas to estimate the burden of disease in Asian countries [42]–[44]. Given the differences in epidemiology and surveillance systems, we think it is more appropriate to rely on studies from the same region.

To implement this approach, we conducted a systematic literature review of articles published in the Web of Science and MEDLINE databases using the keywords “dengue” and “surveillance”; “dengue” and “capture recapture”; or “dengue” and “sensitivity”. We included articles published between 1995 and 2012 in English, Spanish, French, or Portuguese, and obtained a total of 1,676 articles. We then reviewed the titles and abstracts of these articles and found 48 that contained information relevant to the study of EFs for dengue in SEA. We examined these 48 studies plus 14 related articles that we had collected from previous literature reviews in full text, checking references for any additional articles that we could have missed in our search (e.g., national publications not included in international indexes). Eight new articles resulted from reviewing the references.

We then filtered this literature, retaining studies that explicitly reported systematic data on EFs or included the necessary data to estimate them. The specific retention criteria were: (1) use of original, empirical data, (2) implementation of a scientifically valid approach, and (3) the external validity of the data gathered (plausible patterns among age groups, geographic regions, years, and study sites). We complemented the literature review with surveillance data to estimate corresponding EFs [31], [33].

We used original, empirically derived EF_T_ and EF_H_, or the data needed to derive them, where available. Because studies that reported EF used various designs, sampling criteria, methods of analysis, study settings, time frames, among other aspects, for some countries we had to make assumptions about the rate of underreporting in hospitals and/or the share of ambulatory dengue episodes to derive an estimate of EF. We discuss these assumptions further in the next section.

For countries with no original, empirical data, we relied instead on extrapolation of EF_T_ based on quality of health care of each specific country. Because we only had a few empirical observations of EFs, we created a Health Quality Index (HQI) using principal component analysis, including standardized measures of five country-level variables (Table 1) [45], [46]: physicians density per 10,000 population (2005–2010), mortality rate for children <5 years (probability of dying by age 5 per 1,000 live births), neonatal mortality rate (per 1,000 live births), percentage of births attended by skilled health personnel, and total health expenditures per capita (US$). We chose these variables because they were readily available for most countries, and we hypothesized that they represented an underlying factor of healthcare quality. A similar method to address underreporting, based on a measure of accessibility to health care, was used by Murray et al. [47] in recent estimates for burden of disease for 291 diseases and injuries. With dengue being a primarily urban disease, we think that this measure of quality provides a more appropriate measure specifically for dengue. We then extrapolated values of EF_T_ to other countries by running a linear regression with the reporting rates (RR = 1/EF_T_) as the dependent variable and the HQI as an independent variable. Because we only had five observations for EF_T_, we also included two additional empirically-derived estimates from Colombia (EF_T_ = 9.0) [48] and Nicaragua (EF_T_ = 20.3) [40] in the regression, and added a dummy variable to address regional differences. We used RR instead of EF to reduce heteroskedasticity, as suggested by visual inspection of residuals versus fitted values and a substantially lower chi-square in the Breusch-Pagan test. We used RR to predict EF_T_ and confidence intervals from the regressions, and converted the final estimates into EFs for clarity. We only considered empirically-derived EF_T_ and excluded estimates based on expert opinion (i.e., Malaysia) from the regression model.

**Table 1:** Demographic characteristics (2010) and surveillance system of countries in Southeast Asia.

Country	Population (1,000 s)	Urban pop.^a^ (%)	GDP per capita (pc, US$)	Health expenditures (pc US$)	Physician density (10,000 pop)^b^	Child mortality rate <5 yr	Neonatal mortality rate	Skilled attended births (%)	Surveillance system^c^	Quality of surveillance^d^		Lab capability^e^	Reporting site^f^	Reported ages	Peak incidence (ages^g^)
										DF	DHF				
Bhutan	708	30.9	2,010	92	0.2	81	35	51	n.a.	n.a.	n.a.	n.a.	n.a.	n.a.	n.a.
Brunei	401	75.7	28,832	844	14.2	7	3	99	P	+	+ +	S-V	OP&IP	All	25–45
Cambodia	15,053	22.8	791†	42	2.3	89	31	44	P & St	+	+ +	S-V	IP	0–15	5–15
East Timor	1,177	28.1	571†	66	1.0	93	43	19	n.a.	n.a.	n.a.	n.a.	n.a.	n.a.	0–5
Indonesia	232,517	53.7	2,890	57	2.9	41	19	73	P	−	+ + +	S-V	n.a.	All	5–15
Laos	6,436	33.2	976†	40	2.7	61	20	20	P	+	+	S	n.a.	All	5–15
Malaysia	27,914	72.2	8,184	320	9.4	6	3	99	P & A*	++	+ + +	S-V	OP&IP	All	25–45
Myanmar	50,496	33.9	721†	14	4.6	122	48	57	P	−	+ +	S-V	IP	All	5–15
Philippines	93,617	66.4	2,063	67	12.0	32	15	62	P & St	+	+	S-V	OP&IP	All	5–15
Singapore	5,076	100.0	41,893†	1551	18.3	3	1	99	P & A*	+++	+ + +	S-V	OP&IP	All	25–44
Thailand	68,139	34.0	4,850	162	3.0	14	10	99	P	−	+ + +	S-V	IP	All	5–14
Viet Nam	89,029	28.8	1,141†	78	12.2	14	9	88	P & St	+	+ ++	S-V	OP&IP	All	5–14

a Data is an estimation for year 2010, except for Bhutan which corresponds to year 2005.

b Average for 2005–2010, except for East Timor, Philippines, and Thailand which are the average (2000–2009).

c P: Passive surveillance; St: Sentinel surveillance; A: Active surveillance.

d Quality classification: + exists; ++ good; +++ best [6], [24].

e S: serology; V: virology.

f OP: outpatient; IP: inpatient.

g Age category (in years) with peak incidence rate.

† IMF estimate for 2010.

* Gubler reports that both Singapore and Malaysia have active, laboratory based surveillance systems, although Malaysia is mostly passive and has reduced predictive capacity [24]. Both are considered passive systems by Beatty et al. [26].

n.a. denotes data not data not available.

Sources: [24], [26], [31], [36], [38], [45], [46], [54]–[60].

While most underreporting occurs in ambulatory settings, hospitalized episodes are also underreported as indicated by the empirical evidence in the region and elsewhere [16]. The main reason for underreporting in hospitals, even in well-funded health systems such as Singapore, appears to be under diagnosis, which may occur because of limited sensitivity of some diagnostic tests and cost constraints [49]–[51], or because patients are not routinely tested. Other plausible causes include reliance on clinicians' reports [21], particularly in private settings, or limited technical expertise [51]. To estimate EF_H_ for countries where no empirically-derived data were available, we ran a regression with hospitalized dengue RR as the dependent variable and HQI as an independent variable. Because we found no significant correlation (see results for further discussion), we made two assumptions: (i) the rate of underreporting of hospitalized cases was, on average, the same as the average underreporting for countries in the region with empirical data, and (ii) OP∶IP of dengue episodes was, on average, the same as the weighted average of all available empirical studies in the region. While our assumption that EF_H_ is constant for countries with no empirical data ignores idiosyncratic characteristics of health systems, we think that it is a reasonable approach given the relatively low variability we found on underreporting of hospitalized dengue episodes reported in empirical studies in SEA and elsewhere [16]. Assumption (ii) was only necessary for the countries in which reported data came from outpatient and inpatient sources, i.e., Brunei, Laos, and the Philippines. Last, we estimated the annual average of dengue episodes by type of treatment (inpatient and outpatient) and the EF_A_ for each country, where applicable.

### Sensitivity analysis

Because the total cases of dengue remained an uncertainty, we conducted a probabilistic sensitivity analysis, simultaneously varying our parameter estimates based on available information. For countries with empirical data, (1) we estimated the range for EF_T_ using a program evaluation and review technique (PERT) distribution with empirically derived EF_T_ as the best estimate and either the range of empirically derived estimates as the lower and upper bounds where available (Cambodia and Thailand), or alternatively, the 95% prediction interval derived from the regression analysis as the lower and upper bounds (λ = 4 to approximate the shape of a Normal distribution). We used prediction intervals because we were predicting individual EF_T_ for a specific country, and not the expected value of EF_T_ for all subjects, and the standard errors differ in both cases. (2) For Cambodia and Thailand, we varied OP∶IP using a normal distribution based on the weighted average and standard deviation from country-specific studies in different years and/or sites [19], [20], [52]. For Vietnam, Indonesia, Singapore, and Malaysia, for which we did not have enough country-specific OP∶IP observations, we varied EF_H_ using a PERT (λ = 4) distribution with the country-specific empirical estimate (or expert-based estimate for Malaysia) as the best estimate, used 1.0 as the lower bound to be conservative (i.e. all hospitalized episodes of dengue were reported), and the maximum EF_H_ (3.4) from empirical studies among the 12 countries as the upper bound.

For countries where no country-specific empirical data were available, we varied (1) EF_T_ using a normal distribution with μ and σ based on predicted estimates from the regression analysis, (2) EF_H_ using a PERT distribution (λ = 4) with 1 as the lower bound, the average empirical estimate from all 12 countries as the best estimate, and the highest empirical EF_H_ estimate for all countries as the upper bound, and last, (3) OP∶IP using a normal distribution with μ =  weighted average and σ = weighted standard deviation based on all available empirical results from the 12 countries. As an additional sensitivity analysis, we used triangular distributions instead of the PERT distributions to see how the results varied. We computed 20,000 Monte Carlo simulations for each parameter using RiskAMP, version 3.20 [53], which uses the Mersenne Twister random number generator. Iterations drew random values from the distribution of each input. We present results with 95% certainty level bounds.

## Results

### Dengue incidence in selected countries

**Figure 1.Total:**
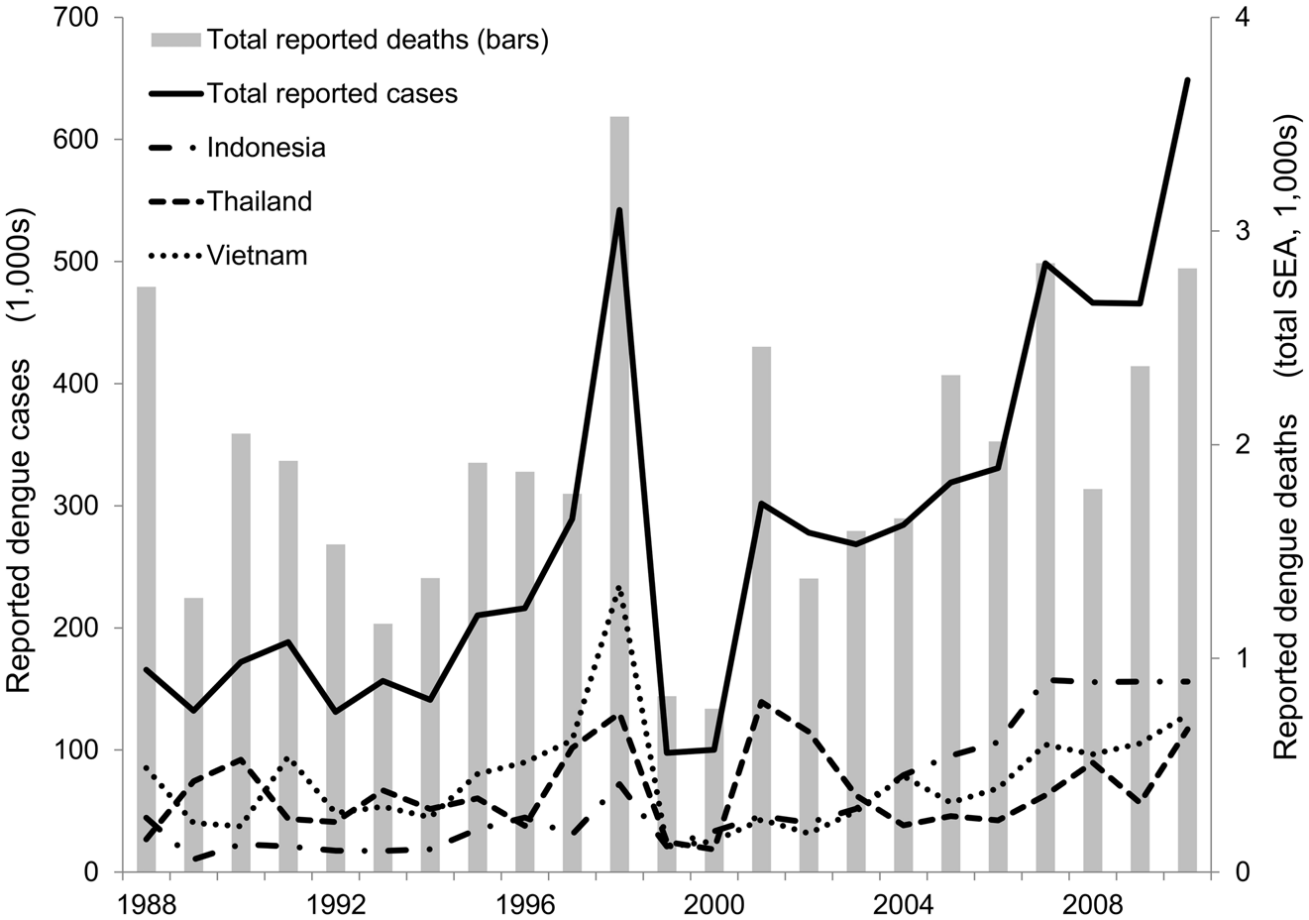
reported dengue episodes in Southeast Asia, 1988–2010. Sources: [11], [13], [29]–[33], [35]–[37].

Figure 1 shows the total dengue episodes and associated deaths reported in SEA from 1988 through 2010.The three countries with the most reported episodes of dengue fever were Viet Nam, Thailand, and Indonesia with cumulative totals of 1.73, 1.54, and 1.43 million cases reported, respectively (1988–2010). Together they represent about 75% of the total reported dengue episodes in the region. In contrast, Bhutan, East Timor, and Brunei, which reported dengue only since 2002, summed 3,358 cases over the same time period. Figure 1 shows the combination of cycles of dengue epidemics in SEA, which peaked in 1998 and 2010 with 540,000 and 650,000 overall reported episodes, respectively, and an increasing trend of total reported episodes that reflects both a growing problem and better reporting. Total reported deaths peaked at 3,500 in 1998, and as expected, were significantly correlated to the total number of dengue episodes (r^2^ = 0.74, p<0.001).

We found considerable variation in surveillance systems in SEA, for example, in the type of dengue case reported by severity, age groups, or type of treatment. Table 1 shows the demographic, health quality, and surveillance system characteristics of the selected countries in SEA [24], [26], [31], [36], [38], [45], [46], [54]–[60].

### Literature review

We identified 11 published articles that reported original, empirically derived EFs or the data needed to derive EFs [17]–[20], [52], [61]–[66], one study based on a systematic two-round Delphi process [67], three empirical studies on dengue burden [43], [68], [69], and seven studies that used EFs based on secondary analysis of published data or exclusively based on expert opinion [42], [44], [54], [70]–[73]. Table 2 shows the main results from the literature review for EFs, or necessary data, in SEA. We extracted data from the articles using a template similar to Table 2, with additional columns (e.g., date the article was reviewed, limitations). We did not consider secondary analysis of data. Although this study is an original research study and not a systematic review, we adapted relevant parts of the PRISMA check list and flowchart to our literature review (Figure S1, Table S1) [74].

**Table 2:** Original, empirically derived expansion factors for countries in Southeast Asia.

Country	Authors	Study years	Study sample	Study site	Methods	Expansion factors (EF) or relevant data	Dengue definition	OP∶IP ratio^b^
*Cohort and capture-recapture studies*								
Cambodia^a^	Wichmann et al. 2011 [19]	2006–2007 (includes epidemic year, 2007)	9,000–10,000 children 0–19 yrs;	Kampong Cham Province: 20 rural and 5 urban villages	Cohort study, active surveillance. Comparison with surveillance data	EF_T_: 9.3; EF_H_: 1.4	WHO clinical case definitions. Lab confirmed dengue.	Weighted OP∶IP in cohort: 7.5∶1
Thailand	Wichmann et al. 2011 [19]	Kamphaeng Phet (KP): 2004–2007(only dengue season); Ratchaburi (R): 2006–2007 (whole year)	KP: 2,000 children 4–13 yrs; R: 3,000 children 3–14 yrs	KP: 11 local primary schools; R: 7 local schools	Cohort studies, active surveillance; comparison with surveillance data	EF_T_: 8.4; EF_H_: 2.9	WHO clinical case definitions. Lab confirmed dengue.	Weighted OP∶IP in cohort: KP: 3.9∶1; R: 0.8∶1; Overall: 2.4
Cambodia	Vong et al. 2010 [20]	2006–2008 (includes epidemic year 2007)	6,700–10,100 participants; 0–19 yrs	Kampong Cham Province: 32 villages and 10 urban areas	Cohort study, active surveillance.	6.9 [Total lab confirmed sympt. cases/total hosp.]	Febrile episode, lab confirmed.; Hospitalized cases <16 yrs are reported.	Weighted OP∶IP in cohort: 5.0∶1
Viet Nam	Tien et al. 2010 [64]	2004–2007 (includes epidemic year 2007)	2,200–3,200 children	Long Xuyen: 3 nursery schools, 2 primary schools, and 1 secondary school	Cohort study, active surveillance. Comparison with surveillance data.	EF_T_: 5.8	Clinical case definition. Lab confirmed dengue.	OP∶IP in cohort: 0.8∶1; asymptomatic to symptomatic ratio:5.3∶1
Thailand	Anderson et al. 2007 [52]	1998–2002 (includes epidemic years 1998, 2001)	2,200 children 5–15 yrs.	Kamphaeng Phet. 12 local primary schools	Cohort study, active surveillance. Lab confirmed dengue	3.4 [lab confirmed dengue/hosp. dengue]; 1.4 [lab. Confirmed dengue/amb. dengue]	Febrile episode, lab confirmed dengue.	OP∶IP in cohort: 3.1∶1
Indonesia	Porter et al. 2005 [63]	08/2000–07/2002	2,500 adults >18 yrs	Bandung, 2 textile factories	Cohort study, active surveillance.	2.3 [lab confirmed dengue/hosp. dengue]; 1.8 [lab confirmed dengue/amb. dengue]	Febrile episode, lab confirmed dengue.	OP∶IP in cohort: 1.3∶1; asymptomatic to symptomatic ratio: 3.1∶1
Cambodia	Vong et al. 2012 [17]	2006–2008 (includes epidemic year 2007)	14,354 individuals <19 yrs	32 villages and 10 urban areas, Kampong Cham^c^	Capture-recapture study. Active surveillance and comparison to reported cases in surveillance system	EF_T_: 16.2; EF_H_: 2,0^d^	Febrile episode, lab confirmed dengue.	OP∶IP in cohort: 7,4∶1
*Hospital and health center surveillance*								
Indonesia	Chairulfatah et al. 2001 [18]	04/1994–03/1995 (includes epidemic year 2005)	650 hospitalized patients with DHF or DSS	4 major hospitals in Bandung	Clinical surveillance for DHF, use of medical records for comparison to reported dengue in surveillance system	EF_H_: 3.3; EF_death_: 2.2 [total deaths from DHF&DSS/reported deaths]	Clinical DHF and DSS	
Viet Nam	Phuong et al. 2006 [62]	4/2001–3/2002	2,100 febrile patients	Binh Thuan Province: 12 community health posts (rural and urban) and one clinic in Phan Thiet (capital)	Clinical surveillance for febrile episodes	5.2 [total lab confirmed dengue/total patients diagnosed with dengue]	Febrile episode, lab confirmed dengue.	
*National surveys*								
Singapore	Yew et al. 2009 [65]	2004 (epidemic year)	4,200 adults >18 yrs	Representative sample of Singapore, from National Health Survey 2004	Representative survey, included blood samples.	23 [lab confirmed dengue infection/reported dengue]	Lab confirmed cases (recent and past infections)	OP∶IP (from Low et al.'s [69]):1.2∶1
*Expert Opinion (Delphi process)*								
Malaysia	Shepard et al. 2012 [67]	2009	National population, combination of various data sources	Not applicable	2-round Delphi process, including experts from private and public sectors, and academia	EF_T_: 3.8; EF_H_:1.7; EF_A_:65.4	Officially reported dengue	OP∶IP derived Delphi process 1.4∶1
*Related studies*								
Cambodia [abstract]	Vong et al. 2007 [61]	Not available	Children <15 yrs (n not available)	Not available	Capture-recapture study. Active surveillance and comparison to reported dengue in surveillance system	EF_T_: 3.1; EF_H_: 2.1	Febrile episode, lab confirmed dengue.	OP∶IP in cohort: 1.2∶1
Thailand [preliminary results]	Endy et al. 2002 [66]	1998–2000	2,200 children 5–15 yrs	Kamphaeng Phet: 12 local primary schools	Cohort study, active surveillance	4.8 [total cases/hospitalized cases]; 1.3 [total cases/ambulatory cases]	Febrile episode, lab confirmed dengue	OP∶IP in cohort: 3.8∶1 Asymptomatic to symptomatic ratio: 1.2∶1

a Cambodia reports only inpatients <16 years.

b Ratio is number of number of dengue cases reported from outpatient (OP) clinics divided by the number from inpatient (IP) or hospitalized settings.

c The study did not include all study villages and urban areas for the entire period.

d EFs were obtained using the weighted averages, based on reported episodes from cohort by year.

Notation: DHF denotes dengue hemorrhagic fever; DSS denotes dengue shock syndrome.

### Estimated EFs by country

As shown in Table 2, we found high quality data to estimate EFs for six countries: Cambodia, Thailand, Viet Nam, Indonesia, Singapore, and Malaysia. Our estimates for Thailand were based solely on cohort studies, and we combined a cohort and a capture-recapture study for Cambodia. We combined cohort studies and clinical surveillance studies to obtain our estimates for Indonesia and Viet Nam. The EF_T_ for Singapore was based on blood samples from a national health survey and EF_H_ was derived by combining these data with reported data from a recent multisite longitudinal study. We used data based on expert opinion to estimate appropriate EFs for Malaysia [67]. While some important studies were not yet available when the expert workshop on dengue reporting in Malaysia took place (e.g., [17], [19]), the country's EF_T_ and EF_H_ were estimated through a rigorous two-round Delphi process (see Figure S2 for details) [75], [76]. Table 3 shows a summary of the parameters used, sources, assumptions, and specific calculations for each country, which we discuss below.

**Table 3:** Parameters used, sources, assumptions, and calculations by country.

Country	Empirical parameters	Sources	Assumptions	Calculations
Cambodia	EF_T_; OP∶IP	[17], [19], [20]	R_H_ = R_T_	N_T_ = EF_T_*R_T_; N_H_ = N_T_/(OP∶IP+1); N_T_ = N_H_+N_A_
Thailand	EF_H_; OP∶IP; R_H_ = 0.79*R_T_	[19], [33], [52], [66]	R_H_ = 0.79*R_T_	R_H_ = R_T_*0.79; R_T_ = R_H_+R_A_; N_H_ = EF_H_* R_H_; N_A_ = N_H_*OP∶IP; N_T_ = N_H_+N_A_; EF_A_ = N_A_/R_A_
Viet Nam	EF_T_; OP∶IP	[19], [20], [52], [63], [64], [68]	OP ∶IP = average empirical OP∶IP	N_T_ = EF_T_*R_T_; N_H_ = N_T_/(OP∶IP+1); N_T_ = N_H_+N_A_
Indonesia	EF_H_; N_T_ = 2.3*N_H_	[18], [63]	R_H_ = R_T_; N_T_ = 2.3*N_H_	N_H_ = EF_H_* R_H_; EF_T_ = 2.3*N_H_/R_T_; N_T_ = EF_T_*R_T_; N_T_ = N_H_+N_A_
Singapore	OP∶IP; 0.565*R_T_ = R_H_; 1/23 infections notified; 18% of infections are symptomatic	[43], [63]–[66], [68], [69]	EF_H_ obtained was too high, so we used instead average empirical EF_H_; 0.565*R_T_ = R_H_; 18% of infections are symptomatic	EF_T_ = 23*0.18; N_T_ = EF_T_*R_T_; R_H_ = 0.565*R_T_; N_H_ = N_T_/(OP∶IP+1); EF_H_ = N_H_/R_H_→too high, i.e. EF_H_ = average; N_T_ = N_H_+N_A_; EF_A_ = N_A_/R_A_
Malaysia	EF_T_, EF_H_, EF_A_	[67]	Explained in [67]	N_T_ = EF_T_*R_T_; N_H_ = EF_H_* R_H_; N_A_ = EF_A_* R_A_
Bhutan, East Timor, Myanmar	EF_T_	Regression estimates	R_H_ = R_T_; EF_H_ = average empirical EF_H_	RR_T_ regression; EF_T_ = (1/RR_T_); N_T_ = EF_T_*R_T_; N_H_ = EF_H_* R_H_; N_T_ = N_H_+N_A_
Brunei, Laos, Philippines	EF_T_	Regression estimates	EF_H_ = average empirical EF_H_ OP∶IP = average empirical OP∶IP	RR_T_ regression; EF_T_ = (1/RR_T_); N_T_ = EF_T_*R_T_; N_H_ = N_T_/(OP∶IP+1); N_T_ = N_H_+N_A_; R_H_ = EF_H_/N_H_; EF_A_ = N_A_/R_A_

Notes: EF_T_ = Expansion factor (EF) total dengue episodes; EF_H_ = EF hospitalized episodes; EF_A_ = EF ambulatory episodes; OP∶IP = outpatient to inpatient ratio of episodes; R_T_ = total reported (R) episodes; R_H_ = hospitalized R episodes; R_A_ = ambulatory R episodes; N_T_ = estimated total episodes; N_H_ = estimated hospitalized episodes; N_A_ = estimated ambulatory episodes; RR_T_ = reporting rate of total episodes of dengue. In the main text the assumptions are numbered as (i) EF_H_ = average of EF_H_ from empirical studies in Cambodia, Thailand, Singapore, Indonesia, and Singapore, and (ii) OP∶IP = average of OP∶IP from available empirical studies [19], [20], [52], [63], [68].

Cambodia reports dengue episodes only among children <15 years old, but approximately 90% of the cases of dengue in Cambodia occur within this group [54], and about 80% occur among children <9 years old [77]. Dengue case definitions are based on WHO guidelines, and do not require laboratory confirmation -only a sample undergo serological or virological testing [77]. Our EF estimates for Cambodia were based on a cohort study [19] and a capture-recapture study [17] both in Kampong Cham province. Because both were carefully designed studies, we obtained EF_H_ and EF_T_ using a weighted average based on total dengue episodes by cohort by year. We also combined Vong et al.'s [20] and Wichmann et al.'s [19] cohort studies and obtained a weighted average of OP∶IP of 6.0∶1. Table 4 shows the estimated EFs and total cases by country. For the sensitivity analysis, we varied EF_T_ using a PERT distribution based on the range of empirical estimates, as stated above, and OP∶IP using a normal distribution with μ = 6.0 and σ = 1.2 – the weighted average and standard deviation based on total dengue cases from both studies. Table 5 shows a summary of the distributions and parameters used in the sensitivity analysis for each country.

**Table 4:** Expansion factors for hospitalized (EF_H_), ambulatory (EF_A_) and total (EF_T_) dengue episodes, and average annual reported and estimated dengue episodes (2001–2010).

						Estimated reported		Estimated total (95% certainty level, sensitivity analysis) a		
Country	EF_H_	EF_A_	EF_T_	Reported total	Sources of reported cases	Hospital	Ambulatory	Total	Hospital	Ambulatory
*EFs based on country-specific empirical studies*										
Cambodia	1.8	n.r.	12.9	14,407	[36], [37]	14,407	n.r.	185,850	26,399	159,451
								(86,508–342,021)	(12,293–48,604)	(74,214–293,417)
Thailand	2.9	29.8	8.5	76,978	[11], [13], [33], [35]	60,813	16,165	657,812	176,357	481,455
								(623,085–831,921)	(166,966–222,926)	(456,119–608,994)
Viet Nam	1.2	n.r.	5.8	76,364	[36], [37]	76,364	n.r.	442,911	81,611	361,300
								(417,578–487,763)	(77,789–176,888)	(265,267–395,092)
Indonesia	3.3	n.r.	7.6	104,457	[11], [13], [29], [35], [37]	104,457	n.r.	792,829	344,708	448,121
								(752,863–932,674)	(215,528–352,837)	(418,376–650,432)
Singapore	2.5^b^	5.0	4.1	6,362	[32], [36], [37]	3,595	2,767	26,339	8,986	17,352
								(14,331–30,256)	(5,426–11,415)	(5,242–22,700)
*EFs based on expert opinion*										
Malaysia	1.7	65.6	3.8	37,866	[30], [31], [36], [37]	36,622	1,244	143,891	62,256	81,635
								(106,427–203,914)	(42,285–101,885)	(25,207–144,506)
*EFs based on data extrapolated from neighboring countries*										
Bhutan	2.5	n.r.	12.9	67	[11], [13], [35]	67	n.r.	866	168	699
								(372–1,366)	(101–213)	(211–1,213)
Brunei	2.5	6.2	4.9	72	[36]	26	46	351	65	286
								(303–399)	(35–209)	(142–339)
East Timor	2.5	n.r.	19	323	[11], [13]	323	n.r.	6,137	808	5,330
								(536–18,150)	(486–1,025)	(255–17,385)
Laos	2.5	56.8	11.3	8,536	[36], [37]	7,116	1,420	96,548	17,790	78,758
								(57,073–135,630)	(8,022–57,748)	(33,625–112,558)
Myanmar	2.5	n.r.	16.2	15,313	[11], [35], [37]	15,313	n.r.	247,943	38,283	209,660
								(35,327–517,378)	(22,882–48,593)	(1,368–481,694)
Philippines	2.5	11.7	7	45,409	[36], [37]	23,283	22,126	315,892	58,207	257,685
								(271,244–360,043)	(31,361–185,358)	(129,613–305,917)
**Total SEA**	2.4	48	7.6	386,154		342,384	43,770	2,917,368	815,636	2,101,732
								(2,722,270–3,378,463)	(715,326–983,735)	(1,871,480–2,534,739)

EFs for the lower panel -based on extrapolations from neighboring countries - were estimated under the following assumptions: (i) EF_H_ was constant and equal to the average EF_H_ of countries in the region for which we had empirical evidence (EF_H_ = 2.5); (ii) to estimate EF_A_ for Bhutan, Laos, and Philippines, we also assumed that the OP∶IP episodes ratio was, on average, constant for these countries and equal to the weighted average from all empirical studies in the region (OP∶IP = 4.4).

n.r. denotes not reported; SEA denotes Southeast Asia.

a The 95% certainty level reported in parentheses was estimated by a probabilistic sensitivity analysis simultaneously varying key parameters in 20,000 Monte Carlo simulations (see Table 5 to see specific parameters and distributions used for each factor in the sensitivity analysis).

b We obtained an empirical estimate for EF_H_ of 3.4 in Singapore; however, given legal requirements and incentives for reporting, we think that this estimate may be too high. The main reason for underreporting of dengue in hospitals seems to be under diagnosis, as patients with undifferentiated fever are not routinely tested, or are tested with serological that may not pick up dengue. An additional factor behind underreporting may be underreporting in the private sector [67], which accounted for about 23 of hospitalizations in Singapore (2009–2011; Ministry of Health Singapore). To be conservative, we used the average for countries with empirical studies (2.5), and used 3.4 as the upper bound in the sensitivity analysis, as shown in Table 5.

**Table 5:** Summary of parameters varied simultaneously in sensitivity analysis and their assumed distributions.

Country	Parameter	Estimate	Distribution^a^	Distribution parameters	Values	Source
*Country-specific empirical studies*						
Cambodia	EF_T_	12.9	PERT	(Min; Best; Max)	(3.9; 12.9; 29.3)	Empirical best estimate, range from empirical studies [17]
	OP∶IP	6.0	Normal	(μ, σ)	(6.0; 1.2)	Weighted average from empirical studies in Cambodia
Thailand	EF_T_	8.5	PERT	(Min; Best; Max)	(8.0; 8.5; 12.5)	Empirical best estimate, range from empirical studies [19]
	OP∶IP	2.7	Normal	(μ, σ)	(2.7; 1.1)	Weighted average from empirical studies in Thailand
Viet Nam	EF_T_	5.8	PERT	(Min; Best; Max)	(5.4; 5.8; 6.7)	Empirical estimate & 95%PI from regression
	EF_H_	1.2	PERT	(Min; Best; Max)	(1.0; 1.2; 3.4)	Empirical estimate, conservative assumption (lower bound) & highest empirical estimate (upper bound)
Indonesia	EF_T_	7.6	PERT	(Min; Best; Max)	(7.1; 7.6; 9.9)	Empirical estimate & 95%PI from regression
	EF_H_	3.3	PERT	(Min; Best; Max)	(1.0; 3.3; 3.4)	Empirical estimate, conservative assumption (lower bound) & highest empirical estimate (upper bound)
Singapore^b^	EF_T_	4.1	PERT	(Min; Best; Max)	(1.0; 4.1; 4.9)	Empirical estimate, conservative assumption (lower bound) & 95%PI from regression (upper bound)
	EF_H_	2.5	PERT	(Min; Best; Max)	(1.0; 2.5; 3.4)	Empirical estimate, conservative assumption (lower bound) & highest empirical estimate (upper bound)
*Based on expert opinion*						
Malaysia^c^	EF_T_	3.8	PERT	(Min; Best; Max)	(2.5; 3.8; 6.2)	Expert opinion (lower bound and best estimate) & 95%PI from regression (upper bound)
	EF_H_	1.7	PERT	(Min; Best; Max)	(1.0; 1.7; 3.4)	Empirical estimate, conservative assumption (lower bound) & highest empirical estimate (upper bound)
*Based data extrapolations from neighboring countries*						
All countries	EF_H_	2.5	PERT	(Min; Best; Max)	(1.0; 2.5; 3.4)	Conservative assumption (lower bound), average (best) & highest empirical estimate (upper bound)
All countries	OP∶IP	4.4	Normal	(μ, σ)	(4.4; 2.2)	Weighted average and standard deviation (s.d.) from empirical estimates
Bhutan	EF_T_	12.9	Normal	(μ, σ)	(12.9; 3.8)	Predicted EF_T_ & s.d. from regression
Brunei	EF_T_	4.9	Normal	(μ, σ)	(4.9; 0.3)	Predicted EF_T_ & s.d. from regression
East Timor	EF_T_	19.0	Normal	(μ, σ)	(19.0; 18.2)	Predicted EF_T_ & s.d. from regression
Laos	EF_T_	11.3	Normal	(μ, σ)	(11.3; 2.3)	Predicted EF_T_ & s.d. from regression
Myanmar	EF_T_	16.2	Normal	(μ, σ)	(16.2; 8.9)	Predicted EF_T_ & s.d. from regression
Philippines	EF_T_	7.0	Normal	(μ, σ)	(7.0; 0.5)	Predicted EF_T_ & s.d. from regression

a We used λ = 4 in all PERT distributions, to approximate the shape of a Normal distribution. We did an additional sensitivity analysis using triangular distributions (lower bound, best estimate, upper bound) instead of PERT distributions.

b The lower bound of the 95% predicted interval for Singapore was truncated at 1.0. Because we are dealing pooled EF_T_ over a series of years, we would expect 1.0 to be the minimum plausible EF_T_. Although it is conceptually possible that EF_T_ might be <1 for a specific region or period of time (e.g., during a dengue outbreak), the reporting in the ambulatory sector is so incomplete that while outbreaks happen periodically, we think it is conservative to assume 1 as a lower bound.

c To be conservative, the lower bound of the PERT distribution for Malaysia is based the lower bound derived from a Delphi process in Malaysia by Shepard et al.[67]. We did not use the lower bound from the 95% CI of the regression (EF_T_ = 5.0) because it is higher than the best estimate available (EF_T_ = 3.8).

Notation: EF_T_ denotes expansion factors for total dengue episodes; EF_H_ denotes expansion factors for hospitalized dengue episodes; OP∶IP denotes outpatient to inpatient ratio; PERT denotes the distribution used in program evaluation and review technique; μ denotes mean;, σ denotes standard deviation; PI denotes prediction interval.

Thailand uses the WHO case definition to report patients of all ages, and laboratory testing is commonly applied to all hospitalized cases. Our estimates for EFs in Thailand were mostly based on Wichmann et al.'s study [19] but we refined their estimates using data from a previous cohort study (1998–2002) [52], [66]. Wichmann et al. compared dengue incidence in the cohort to reporting data from the national surveillance dataset, stratifying data by type of management (inpatient and outpatient), year, and age group, and estimated an average EF_H_ of 2.9, and OP∶IP of 2.5∶1. Using these estimates, the authors derived an EF_T_ of 8.4. Another robust 1998–2002 cohort study in Kamphaeng Phet [52], [66] provided an estimate of OP∶IP. A weighted average based on dengue episodes by year between these studies gave us an OP∶IP of 2.7∶1. Using detailed surveillance data on reported cases for years 2003–2009 which suggests that on average 79% of reported dengue corresponds to hospitalized cases [33], we adjusted Wichmann's [19] EFs. We derived an EF_A_ = 29.8 and EF_T_ = 8.5 (Table 4). For the sensitivity analysis, we varied OP∶IP using the weighted average and standard deviation based on total dengue episodes reported by Anderson et al. [52] and Wichmann et al. [19], and EF_T_ using a PERT distribution based the range of empirically derived estimates [19] (Table 5).

We obtained EF_T_ for Viet Nam based on a children cohort study with active surveillance in Lon Xuyen (2004–2007) [64]. Tien et al. compared the average annual incidence rate of laboratory-confirmed dengue in the cohort with incidence data obtained from the national surveillance system for the same years, age groups, and region. Using the reported data, we estimated an average EF_T_ = 5.8. Until 2005, only DHF and DSS were reported in Viet Nam; hence, we assumed that most reported episodes of dengue were hospitalized. Tien et al. reported an average rate of OP∶IP of suspected dengue of 0.8. While the OP∶IP ratio in Viet Nam is probably lower than in other countries because hospitalization is required for all children with suspected dengue [64], the very high proportion of cases that were hospitalized might have been an artifact of the study's procedures (a prospective children cohort adjacent to the provincial hospital). Instead, we used the weighted average OP∶IP for all studies in the region (4.4) and obtained an EF_H_ = 1.2. Using active surveillance, Phuong et al. [62] found that there were 5.2 serologically confirmed dengue episodes for each patient diagnosed with dengue -although the accuracy of dengue diagnosis was less than 50%. This number provides an external validation of our EF_T_ estimate for Viet Nam, since if all cases diagnosed were reported – which is not likely the case – we would expect about 5.2 laboratory-confirmed episodes of dengue for each reported episode.

The EFs for Indonesia were based on two empirical studies [18], [63]. Reporting DHF episodes within 24 hours following diagnosis is required by law in Indonesia, and Chairulfatah et al. [18] found 3.3 hospitalized episodes of DHF for each reported episode, with 50% of cases >14 years. Because the accuracy of diagnosis increases with severity [64], we would expect DHF episodes to be reported more frequently than only acute dengue inpatient episodes, so we believe our estimate is rather conservative. Considering that Indonesia only reported inpatient dengue episodes, we combined this EF_H_ with Porter et al.'s estimate of 2.3 episodes of dengue for every hospitalized episode [63], and obtained an EF_T_ of 7.6.

We estimated EF_T_ for Singapore mainly based on an empirical study by Yew et al. [65]. Yew et al. found evidence suggesting that only one out of 23 dengue infections (including symptomatic and asymptomatic) were notified. Because Yew et al. did not provide information on the ratio of asymptomatic to symptomatic cases of dengue infection in Singapore, we obtained this ratio from a weighted average based on the total number of dengue infections by cohort-year from cohort studies in Indonesia, Thailand, and Viet Nam [63], [64], [66]. On average, 18% of dengue infections were symptomatic, so we derived an EF_T_ of 4.1. We estimated EF_H_ using the OP∶IP (1.16∶1) ratio derived from data reported by Low et al. from the multicenter longitudinal Early Dengue Infection and Outcome Study (EDEN) in Singapore, 2005–2010 [68], [69]. Last, we obtained from Carrasco et al. [43] that 56.5% of the total dengue episodes reported to the surveillance system were hospitalized patients. Carrasco et al. obtained this proportion using data from the Communicable Diseases Division of the MoH, the EDEN study, and the Adult Retrospective Dengue Study at Tan Tock Seng Hospital (ARDENT). From these estimates, we derived an EF_H_ of 3.4, and an EF_A_ of 5.0. Singapore has strict legal requirements and incentives for reporting dengue, and a high quality surveillance system. Thus, an EF_H_ of 3.4 may be an overestimate of underreporting. To be conservative, we used instead an EF_H_ of 2.5, equivalent to the average EF_H_ from empirical studies as our best estimate and 3.4 as the upper bound in the sensitivity analysis. For the same reasons, we also used 1.0 as the lower bound for both EF_H_ and EF_T_ in the sensitivity analysis.

We obtained the EFs for Malaysia from a recent study by Shepard et al. [67] that combined multiple data sources to refine the estimates of underreporting of dengue cases, including data from the MoH, private laboratories, previous literature, and a two-round Delphi process (Figure S1). The first round of the Delphi process took place during a workshop in Malaysia, where evidence was discussed among experts from public and private sectors, and academia. The second round was conducted some weeks later among the same group after analyzing results from the workshop, updating evidence, and adjusting the results for internal consistency. The results from the Delphi process suggested an EF_T_ = 3.8, EF_H_ = 1.7, and an EF_A_ = 65.6. The estimated EFs were conservative, since some important studies were not yet available for either round (e.g., [17], [19]). Shepard et al. obtained a distribution of dengue episodes by type of treatment using 2009 data, which was used to update their estimates using the average reported cases in 2001–2010. We think these EF estimates were as accurate as available evidence allowed at the time the Delphi panel took place. We varied EF_T_ and EF_H_ for Vietnam, Indonesia, Singapore, and Malaysia in the sensitivity analysis, as shown in Table 5.

### EFs for hospitalized and total cases based on data extrapolation

**Figure 2:**
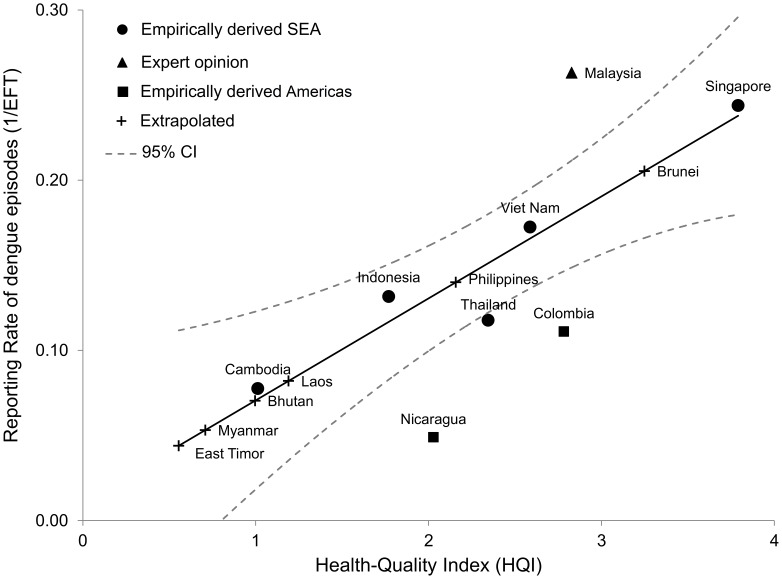
Empirical and predicted reporting rates for total dengue and Health Quality Index in Southeast Asia and the Americas. Source: Authors' calculations from [17]–[20], [24], [26], [31], [36], [38], [40], [45], [46], [48], [53]–[67].

We extrapolated EF_T_ based on the country's quality of health care, defined by HQI, for countries where no country-specific empirical data were available. The five standardized country-level variables were internally consistent (Chronbach's alpha: 0.92; Kaiser-Meyer-Olkin measure >0.68 for all variables) and loaded to a single factor that accounted for most of the variability of the data (Eigenvalue: 3.8). Figure 2 shows the empirically derived reporting rates by country and the regression results with a 95% confidence interval (R^2^ = 0.93, HQI significant at p<0.01) for SEA. We predicted EF_T_ for countries where no empirical data was available based on these regression results (Table 4). To check robustness, we also ran the regressions using estimates for countries in SEA only, and obtained similar results (R^2^ = 0.91, HQI significant at p = 0.01). Regression results were also similar using EF_T_ as the dependent variable in the regression specification (all countries: R^2^ = 0.74, HQI significant at p = 0.08; only countries in SEA: R^2^ = 0.83, HQI significant at p = 0.03).

We found no significant correlation between HQI and EF_H_, which is possibly explained by the relatively low variability of underreporting of hospitalized dengue episodes and the few observations available. The average EF_H_ for countries in SEA with empirical data was 2.5, which was within the range of EF_H_ estimates obtained from systematic empirical studies in Puerto Rico [78], [79] (all episodes 2.4; DHF: 2.9) and Brazil [39] (1.6). We also obtained a weighted OP∶IP average of 4.4∶1 [19], [20], [52], [63], [64], [68].

Based on assumption (i) EF_H_ = 2.5, we directly derived the average distribution of dengue episodes in 2001–2010 for Bhutan, East Timor, and Myanmar. Considering both assumptions, (i) EF_H_ = 2.5 and (ii) OP∶IP = 4.4∶1, we estimated EF_A_ and the distribution of dengue episodes by treatment for the remaining three countries: Brunei, Laos, and Philippines. Table 4 shows a summary of the results: EFs by country and the average annual reported and estimated total dengue episodes by type of treatment (2001–2010).

Overall, there were on average 386,154 annual dengue episodes reported in SEA from 2001 through 2010. Using our expansion factors, we projected that a total of 2,917,368 symptomatic dengue episodes (95% certainty level: 2,722,270–3,378,463; interquartile range: 2,915,658–3,149,257) occurring each year on average, of which 815,636 were hospitalized (95% certainty level: 715,326–983,735) and 2,101,732 ambulatory (95% certainty level: 1,871,480–2,534,739) episodes. We obtained an overall EF_T_ in the region of 7.6 (95% certainty level: 7.0–8.8) dengue episodes for every reported episode.

Last, as an additional sensitivity analysis, we did 20,000 Monte Carlo simulations using triangular distributions instead of PERT distributions, maintaining the same lower and upper bounds and best estimate for EFs. The results from were very similar. We obtained a 95% certainty level of 2,498,726–3,513,599 total dengue cases (interquartile range: 3,012,551–3,265,965), 676,098–1,023,528 hospitalized cases, and 1,930,568–2,668,726 ambulatory cases. The 95% certainty level of overall EF_T_ was 7.2–9.1.

## Discussion

Obtaining an accurate estimate of the total number of episodes is a critical step in the study of the disease and economic burden of dengue. Our analysis suggested that there is substantial underreporting of symptomatic dengue illness in SEA, with an average of only about 13.2% (95% certainty level: 11.4%–14.3%) of all symptomatic dengue episodes reported to surveillance systems. Under-reporting is particularly a problem during inter-epidemic periods, while over-reporting (or substantially less under-reporting) might occur during epidemics. Undifferentiated fever due to dengue is indistinguishable for other viral fevers and even for DHF the differential diagnoses are very broad in the early febrile phase. But on balance, the overall effect is for all dengue to be underreported as evidenced by active surveillance studies of dengue in the region. We estimated a total of about 2.9 million annual dengue episodes occurring in 12 countries in SEA (2001–2010), with an average ratio of OP∶IP of 2.6 (95% certainty level: 2.0–3.3), which represent a serious burden to healthcare systems in the region.

The strengths of our approach include our systematic procedures to identifying high quality empirical studies on EFs in SEA, our systematic inclusion of all the studies that met these standards, and our adjustment for the most salient site-level characteristics. In implementing our study, we conducted a systematic literature review and filtered the studies based on specific criteria. The resulting empirical EFs reflect the behavior of patients, health professionals, and the laboratory and public health systems in diagnosing, treating and documenting dengue. Because EFs are ratios of two measures (projected and reported numbers of cases), they are more robust than raw numbers. High and low rates of dengue incidence tend to raise or lower both projected and reported numbers, without necessarily affecting the EF. The resulting empirical total EFs were relatively consistent, varying by a factor of only 3.1 from the lowest (4.1) to highest value (12.9) across all the countries. These similarities may reflect the health professionals shared experiences in training, professional conferences, publications, and guidance from the World Health Organization across SE Asia. Finally, while past studies have documented variations among dengue surveillance systems [6], [24], [26], [38], [80], partly summarized on Table 1, we were able to control for an important part of this variation. Because the completeness of dengue reporting was expected to reflect, in part, the quality of the health system overall, we controlled for this variation using a HQI. We expected higher quality health systems to have better reporting, and our regression results were consistent with these expectations. A relevant aspect of our estimates for total episodes of dengue is that they tend to smooth out geographic and time variation, since we used averages of reported cases in the last decade (2001–2010) and, in many cases, used weighted averages for parameters across studies and years. Even though our annual estimates do not reflect the actual idiosyncratic variability of total symptomatic dengue infections, they provide a more stable estimate of dengue burden in the region.

While we believe our methods provide a reasonable empirical basis to estimate EFs and the total symptomatic episodes of dengue in SEA, several limitations must be acknowledged. First, the number of published empirical studies in SEA for estimating the total expansion factor is limited -only 11. These studies varied in their methodologies (e.g., cohort studies, capture-recapture, hospital and health center surveillance, national surveys), age groups considered (e.g., cohorts of adult workers versus cohorts of children), types of study sites (e.g. major public hospitals, rural health posts), or severity of dengue infection reported (e.g., dengue fever, DHF, DSS). Similarly, our regression relating RR to HQI was also based only on our best estimates for five countries in SEA and two in the Americas. When there is little information on a subject, each new piece can make a considerable difference. For example, the new available evidence estimating EF_T_ for Malaysia, based on expert opinion, seems to be conservative [67]. As more empirical studies on underreporting become available, and surveillance systems improve their efficacy, EF estimates will be more accurate.

Second, the specific study locations and age groups were generally ones to which the researchers had access. They were generally not randomly selected and are not necessarily representative of the country as a whole. For example, the specific studies in some countries may suggest a lower proportion of ambulatory cases relative to hospitalized cases, as might be the case of Indonesia, were the OP∶IP of 1.3 may be explained because textile workers in Bandung [63] probably have higher income and better healthcare access than the average person in that country. We also found other empirical studies where this ratio may seem too high [19], [81].

Third, to the extent that site-to-site variation remains a factor, our ability to adjust for it was limited to our single variable, HQI. Also, the range we used for EF_T_ in the sensitivity analysis considered only between-country and not within-country heterogeneity, which resulted in narrower ranges for our estimates of total episodes of dengue than if we had been able to include within-country heterogeneity.

Fourth, we found no empirical or high quality studies for six of the 12 countries included in our estimates of dengue episodes. We addressed this constraint by extrapolating data from countries with empirical studies, but could not control for other factors, such as characteristics of national healthcare systems, the level of dengue awareness, or various other relevant factors, such as virus serotypes, rainfall, or global commerce and tourism [82]. As expected, the 95% certainty level for overall EF_T_ in countries with empirical studies (6.8–8.1) was much narrower than for countries where we extrapolated EF_T_ (6.4–13.6).

Fifth, some evidence suggests that the rate of underreporting varies by the severity of dengue symptoms, with reporting increasing for more severe dengue [39]. This evidence may imply that the most modest cases would have the highest degree of underreporting. Due to data limitations, we were not able to categorize numbers of reported cases by dengue severity. Despite all these limitations, we believe that adjustment for underreporting of dengue is critical to estimate the true economic burden, and we sought to make the best adjustments possible with available data.

Estimating the rate of underreporting of dengue with accuracy is a very complex task, particularly distinguishing the factors that drive underreporting to surveillance systems. More accurate estimates of the rate of underreporting of dengue would require a better understanding of the epidemiology of dengue. We think that long-term nationally representative cohort studies that could factor in a wide range of variables related to healthcare systems (e.g., facility types, public and private sectors, number of physicians, specific lab tests used, dengue definition, diagnosis, healthcare access and coverage), geography (e.g., rural and urban population, altitude, latitude, rainfall), virus (e.g., dengue serotypes and genotypes), vectors (e.g., vector control activities, public awareness campaigns), and dengue sequelae (e.g., severity of disease, long-term symptoms, duration and intensity) would be the ideal source of data. However such studies require considerably more time and resources than alternative designs, such as regional or local cohort studies, capture-recapture studies, or Delphi panels.

By generating better estimates of EFs, this paper will contribute towards a better understanding of underreporting of dengue episodes and improving regional and country-specific estimates of the economic and disease burden of dengue in SEA [83]. While this study was focused on dengue in SEA, analogous principles apply to other regions of the world and other diseases reported through health information and surveillance systems. Estimating EFs is an important middle step towards estimating the economic burden of disease, and the cost-effectiveness of vaccines and other preventive and curative approaches.

## Supporting Information

Figure S1 **PRISMA 2009 Flow Diagram.** Source: [74].
(TIF)

Figure S2 **Description of a Delphi Process.** Note: A Delphi process is designed to use expert knowledge systematically to help solve complex issues when there are insufficient data. It aims to get the most reliable expert opinion through several rounds of consultation with controlled opinion feedback. Since the Delphi method is based on experts with a range of field experience, discussion and opinions strengthen the grounding of the results which will be more robust with diverse contexts and settings. The Delphi method usually leads to independent thought and gradual formation of a considered opinion, as experts have the opportunity to revise individual views based on available evidence and other factors the expert might have overlooked on a previous round. Sources: [75], [76].
(TIF)

Table S1 **PRISMA checklist for literature review.** Note: As this manuscript is not a systematic review nor meta-analysis, the entries in the checklist are limited to those items applicable to this manuscript. Source: [74].
(DOCX)
